# A rare case of a chest wall abscess caused by a migrating oesophageal stent. An iatrogenic gastrocutaneous fistula

**DOI:** 10.1259/bjrcr.20160138

**Published:** 2017-03-30

**Authors:** Ravindran Karthigan, Matthew Townsend, Nathan Chan, Husein Kaderbhai, Yasmin Tabbakh, Antonio Leyte Golpe, Branavan Rudran, Christopher Hadjittofi, Sameer Zar, Dimitrios Pissas, Kashif Burney

**Affiliations:** ^1^Department of Radiology and General Surgical , Epsom and St Helier’s NHS Trust, Epsom, England, UK; ^2^Department of Anatomy and Human Sciences, King's College, University of London, London, United Kingdom

## Abstract

We report the case of a 65-year-old male, who presented with septicaemia and a chest wall mass on a background of oesophageal carcinoma. This chest wall mass measured 10 cm by 10 cm, was fluctuant, and was situated on the anterior chest wall. Owing to local erythema and surgical emphysema, necrotising fasciitis was suspected and thus intravenous antibiotic and fluid therapy were instituted. Following a chest radiograph, which confirmed the presence of subcutaneous gas, the patient underwent thoraco-abdomino-pelvic CT, which demonstrated oesophageal stent migration through the gastric fundus to the chest wall, between the 10th and 11th left ribs. Through this migration tract, the chest wall was contaminated with gastric contents, accounting for the mass and sepsis. The patient underwent endoscopic stent removal, and incision and drainage to create a gastrocutaneous fistula. Additionally, a nasojejunal tube and intravenous line were sited for jejunal and total parenteral nutrition, respectively, in order to promote healing of the fistula.

## Clinical presentation

A 65-year-old male presented to the Emergency Department with tachycardia (100 beats per minute), hypotension (100/60 mmHg) and high-grade pyrexia (38.0°C). His medical history was notable for oesophageal carcinoma, treated by oesophageal stent placement. Physical examination revealed an 10 × 10 cm erythematous, fluctuant, warm mass over the left anterior chest wall with associated surgical emphysema. Initial investigations were remarkable for leukocytosis and elevated CRP levels.

**Figure 1.**
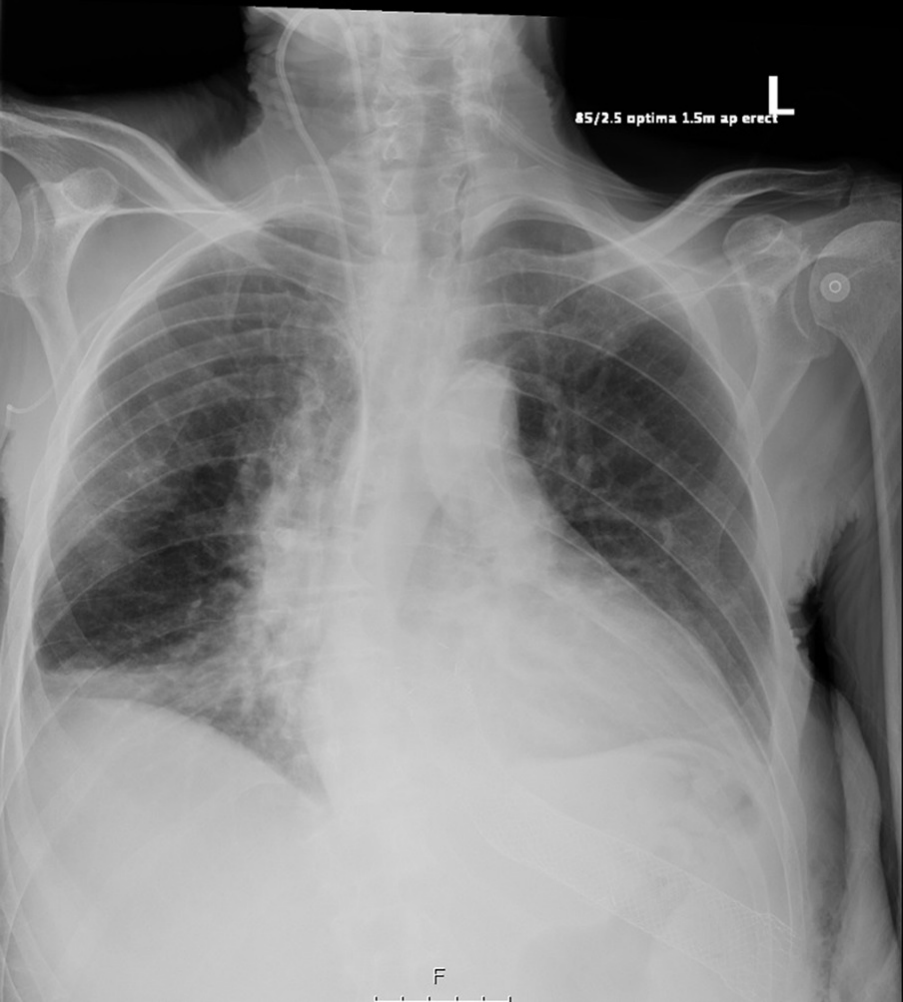
AP erect portable chest X-ray showing right lower lobe consolidation. A right internal jugular line. More importantly the oesophageal stent has the distal end at the level of the ribs, with gas in the subcutaneous fat adjacent to it.

**Figure 2.**
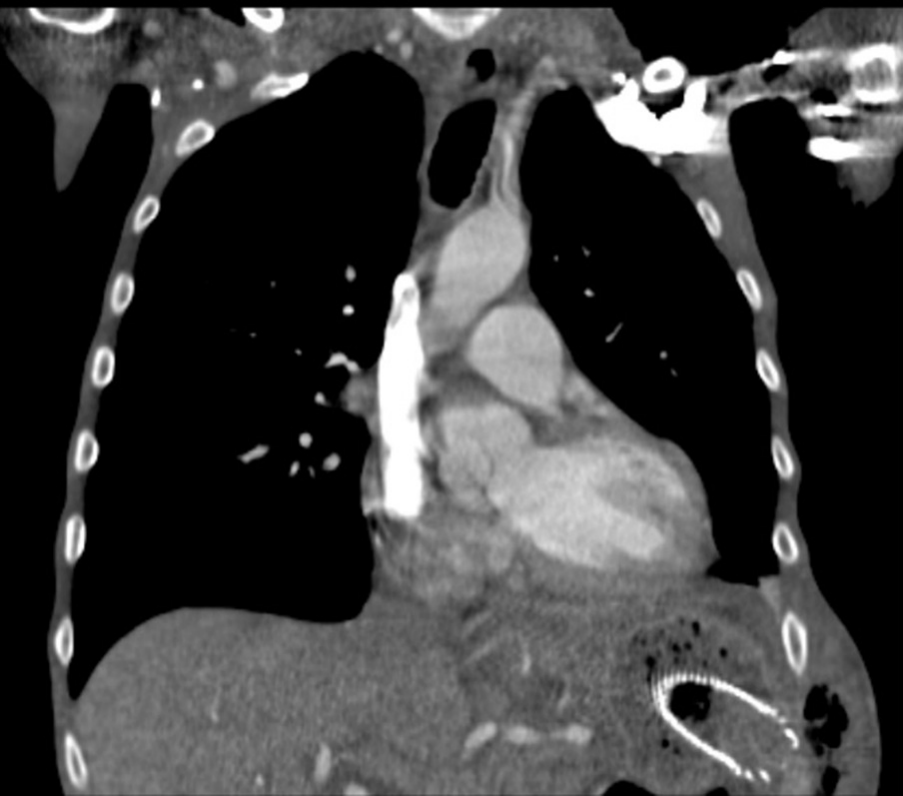
A coronal slice of the CT thorax of the patient showing inferior migration of the oesophageal stent, which has gone through the greater curvature of the stomach, perforating the stomach wall and extending into the lateral aspect of the chest wall. There is also surgical emphysema with soft tissue swelling on the left lateral inferior chest wall and left upper lateral abdominal wall.

**Figure 3.**
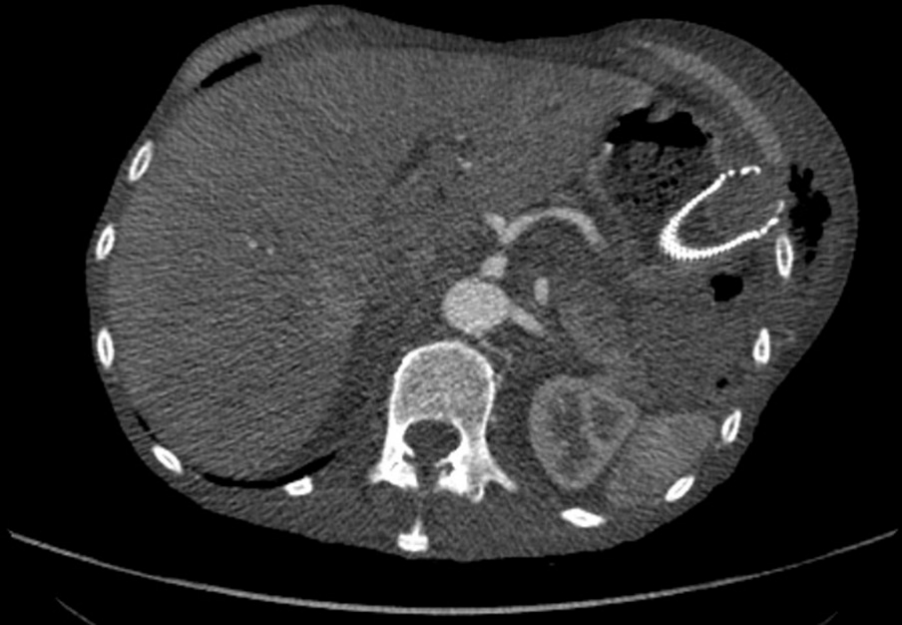
A transverse slice of the same CT scan showing the migration of the oesophageal stent in another plane. Demonstrating the extend of the tissue swelling and the amount of gas in the subcutaneous tissue.

Given the above, necrotising fasciitis was suspected and intravenous antibiotic and fluid therapy were instituted. The patient was admitted under the care of the General Surgery team, and underwent a chest radiograph, which confirmed the presence of gas in the left anterior chest wall (Figure 1). Subsequent thoraco-abdomino-pelvic CT revealed oesophageal stent migration through the gastric fundus to the chest wall between the 10th and 11th left ribs (Figures 2,3), which led to chest wall contamination by gastric contents.

## Treatment

**Figure 4.**
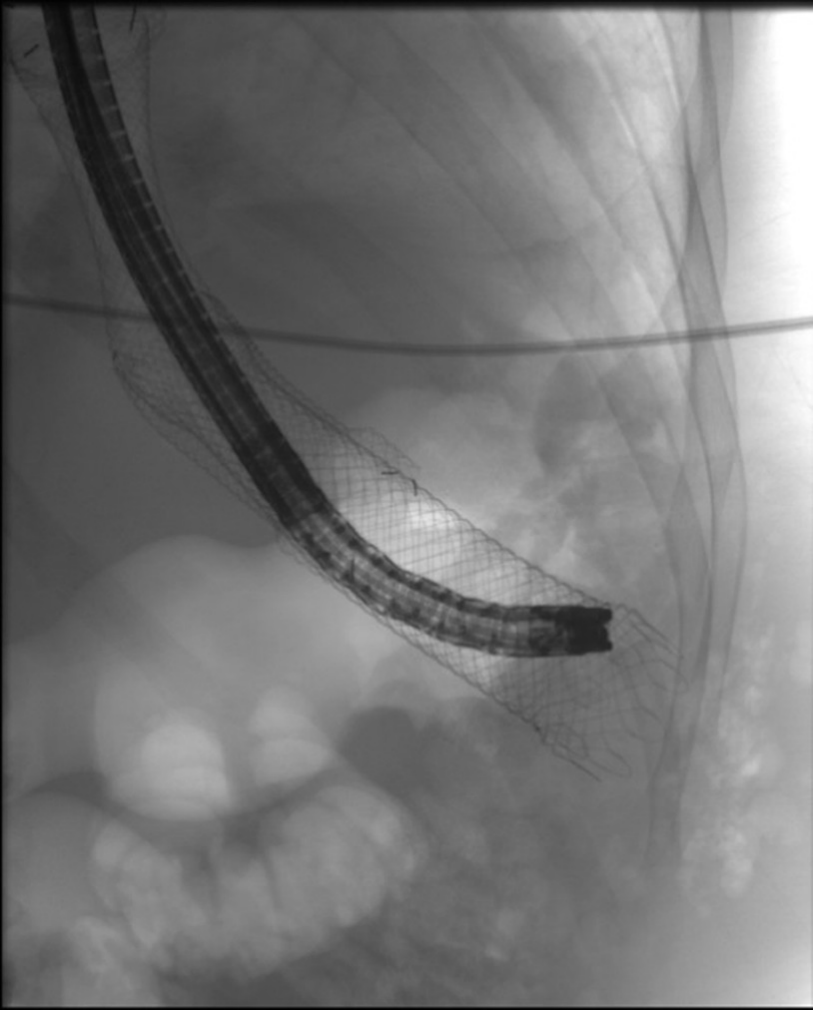
A picture of the endoscopic removal of the migrated stent which was carefully removed under fluoroscopic guidance.

**Figure 5.**
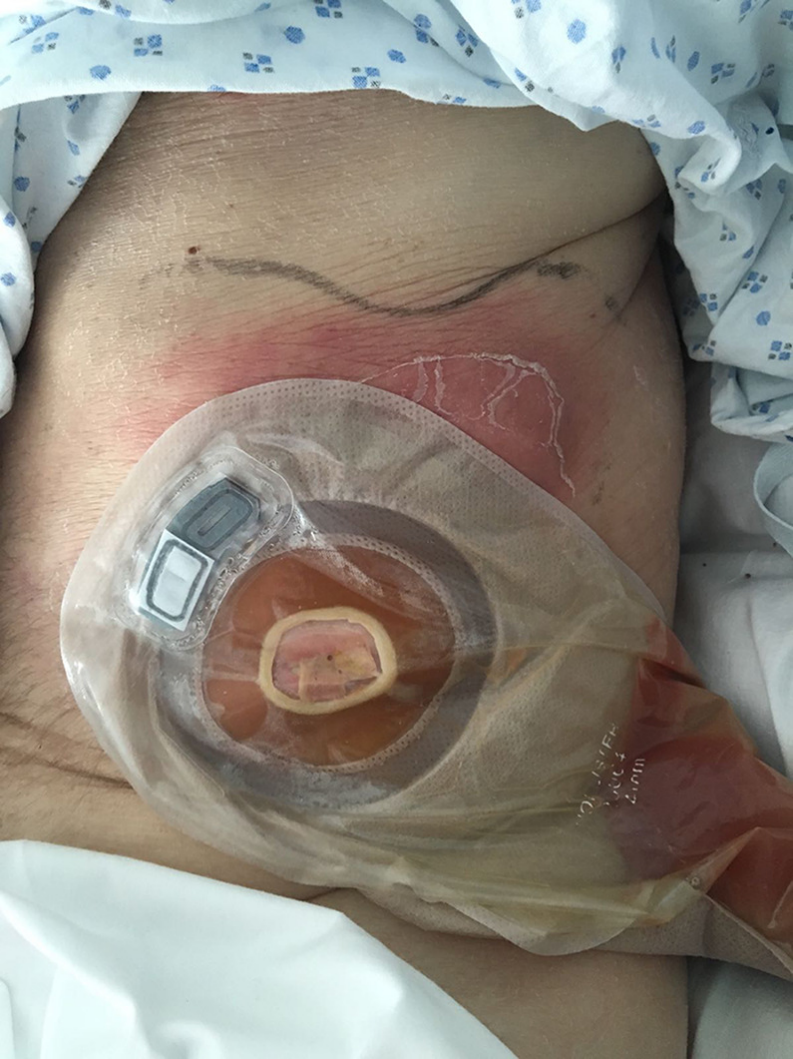
A picture of the chest wall following incision and drainage of the abscess caused by the migration of the stent. A corrugated drain is seen leaving the fistulae patient between the stomach and the stoma bag.

Following a multidisciplinary team discussion, the stent was removed endoscopically (Figure 4), and chest wall mass was incised and drained under general anaesthesia, with corrugated drain insertion and stoma bag application (Figure 5), thus creating a controlled gastrocutaneous fistula. From this point onwards, nutrition was provided via a nasojejunal tube, as well as intravenously.

## Outcome, follow-up and discussion

Necrotising fasciitis is a surgical emergency and should remain the primary differential diagnosis in patients with sepsis, skin changes and surgical emphysema. Not until this has been excluded should alternative diagnoses be considered.

Stent migration is a recognized complication of endoscopically placed metal stents for malignant oesophageal stricture.^1^ Although trachea-oesophageal and aorto- oesophageal fistulation is well described in the literature, gastrocutaneous fistulas are rare, accounting for only 0.5–3.9% of patients who undergo gastric surgery.^2^ They usually occur following iatrogenic gastric injury, breakdown of gastroenteric anastomosis or failure of a healing gastrostomy tube tract. However, to the best of our knowledge, gastrocutaneous fistulation following distal oesophageal stent migration has not been previously reported.

This case highlights the importance of imaging, both in establishing a diagnosis and in planning for definitive treatment. In patients with advanced malignancies, who may be treated palliatively, the benefit of invasive intervention must always be balanced with the risk of complications.

Fistulas can be managed using a “SNAP” approach—sepsis, nutrition, anatomy and proceed. These same principles can be applied in all cases, even in cases where site or mechanism of fistulation are rare.

Sepsis management involves medical treatment, as well as mechanical/surgical source control. This patient was treated initially with intravenous antibiotics, but it was not until incision and drainage of his chest wall had been performed that source control was obtained. Strict nil-by-mouth precautions and use of a proton pump inhibitor limited further passage of gastric contents into the subcutaneous tissues.

Pre-operative nutritional optimization is paramount for successful outcomes, either as enteral nutrition introduced distal to the fistula, or in the form of total parenteral feeding. This is essential to prevent further physiological deterioration of a malnourished patient, to rest the gastrointestinal tract and facilitate healing of the fistula. This patient commenced total parenteral nutrition early in his admission, supplemented by nasojejunal feeding.

Detailed anatomy of a fistula is often hard to determine and interpret. A review of radiographic imaging techniques has shown that contrast studies, small bowel follow-through, CT, MRI and fistulography have all shown success in demonstrating enterocutaneous fistulae.^3^ The role of radiology extends beyond pure anatomical definition to include diagnosis of associated processes and even provision of therapeutic alternatives to treatment such as guided percutaneous drainage. The advantage of cross-sectional imaging techniques over conventional contrast studies includes the additional visualization of extraluminal disease and inflammation. Intravenous contrast is used to enhance the identification of these associated processes, and oral contrast can also be used in cases to differentiate bowel loops from extraluminal collections and abscesses. Identification of a high proximal location of the fistula within the gastrointestinal tract is important in predicting nutritional and fluid requirements.

In this case, contrast-enhanced CT of the thorax and abdomen provided adequate characterization of the fistula. This information should be reviewed by a multidisciplinary team including radiologists and surgeons, to allow detailed planning and early intervention.

Gastrocutaneous fistulas resolve spontaneously in 6% of benign cases.^4^ However, this does not apply in cases where sepsis or foreign bodies are present, which can prolong tract patency. The decision for surgical intervention, either via open or laparoscopic techniques is twofold; to facilitate control of sepsis, and management of the gastric leak. Conservative management is an option and should be considered in haemodynamically stable patients without signs of sepsis. The emphasis here lies in maintaining adequate nutrition, electrolyte balance and managing infection.

The procedures described is this case involved endoscopic removal of the migrated stent, as well as percutaneous drainage of the chest wall abscess. The creation of this iatrogenic gastrocutaneous fistula allowed subsequent source control of infection and output monitoring.

In summary, this case represents the first report of a rare complication of oesophageal stent placement for malignant disease. Furthermore, it highlights the fact that even rare variations of well-described pathology can be safely and effectively managed through the application of established general surgical principles.

## Learning points

1. An erythematous mass with surgical emphysema should be assumed to be necrotising fasciitis until proven otherwise.
2. Stent migration is a rare and potentially life-threatening complication.
3. Regarding management of fistulae, it takes a multidisciplinary team decision and carefully planning for surgery, nutrition and to consider the patients best interest.

## Consent

Written informed consent for the case to be published (including images, case history and data) was obtained from the patient(s) for publication of this case report, including accompanying images.
